# Perceived Need for Psychosocial Support After Aortic Dissection: Cross-Sectional Survey

**DOI:** 10.2196/15447

**Published:** 2020-07-06

**Authors:** Gunther Meinlschmidt, Denis Berdajs, Roger Moser-Starck, Alexander Frick, Sebastian Gross, Ulrich Schurr, Friedrich S Eckstein, Sabina Hunziker, Rainer Schaefert

**Affiliations:** 1 Department of Psychosomatic Medicine Faculty of Medicine University of Basel and University Hospital Basel Basel Switzerland; 2 Division of Clinical Psychology and Epidemiology Department of Psychology University of Basel Basel Switzerland; 3 Division of Clinical Psychology and Cognitive Behavioral Therapy International Psychoanalytic University Berlin Germany; 4 Division of Cardiac Surgery University Hospital Basel Basel Switzerland; 5 Division of Medical Communication Department of Psychosomatic Medicine University Hospital Basel Basel Switzerland

**Keywords:** aortic dissection, patient involvement, psychosocial support, psychosomatic, psychotherapy, treatment need

## Abstract

**Background:**

The gold standard management of aortic dissection, a life-threatening condition, includes multidisciplinary approaches. Although mental distress following aortic dissection is common, evidence-based psychosocial interventions for aortic dissection survivors are lacking.

**Objective:**

The aim of this study is to identify the perceived psychosocial needs of aortic dissection survivors by surveying patients, their relatives, and health professionals to inform the development of such interventions.

**Methods:**

This study used a cross-sectional survey and collected responses from 41 participants (27 patients with aortic dissection, 8 relatives of patients with aortic dissection, and 6 health professionals) on key topics, types of interventions, best timing, anticipated success, and the intended effects and side effects of psychosocial interventions after aortic dissection.

**Results:**

The principal intervention topics were “changes in everyday life” (28/41, 68%, 95% CI 54.5%-82.9%), “anxiety” (25/41, 61%, 95% CI 46.2%-76.2%), “uncertainty” (24/41, 59%, 95% CI 42.9%-73.2%), “tension/distress” (24/41, 59%, 95% CI 43.9%-73.8%), and “trust in the body” (21/41, 51%, 95% CI 35.9%-67.5%). The most commonly indicated intervention types were “family/relative therapy” (21/41, 51%, 95% CI 35%-65.9%) and “anxiety treatment” (21/41, 51%, 95% CI 35%-67.5%). The most recommended intervention timing was “during inpatient rehabilitation” (26/41, 63%, 95% CI 47.6%-77.5%) followed by “shortly after inpatient rehabilitation” (20/41, 49%, 95% CI 32.4%-65%). More than 95% (39/41) of respondents anticipated a benefit from psychosocial interventions following aortic dissection dissection, expecting a probable improvement in 68.6% (95% CI 61.4%-76.2%) of aortic dissection survivors, a worse outcome for 5% (95% CI 2.9%-7.9%), and that 6% (95% CI 1.8%-10.4%) would have negative side effects due to such interventions.

**Conclusions:**

Our findings highlight a substantial need for psychosocial interventions in aortic dissection survivors and indicate that such interventions would be a success. They provide a basis for the development and evaluation of interventions as part of state-of-the-art aortic dissection management.

## Introduction

Aortic dissection is a rare but life-threatening condition. The annual incidence of the condition in older adults is up to 35 cases per 100,000 people, with a slight male preponderance [1-3]. This low incidence explains the delayed diagnosis; only 39% of patients are diagnosed within 24 hours after symptom onset [4]. Prognosis is grave, with a lethality rate of 1% to 2% per hour after onset of symptoms in untreated patients [5]. Preadmission mortality is 20% [6]. Operative 30-day mortality for ascending aortic dissection at experienced centers is still between 10% and 35% [4]. In a propensity-matched retrospective analysis, survival rates in patients with acute type A dissection were 91% after 30 days, 74% after 1 year, and 63% after 5 years [4]. Furthermore, 10-year survival rates of patients who are discharged from hospital range from 30% to 60% [6]. The underlying pathophysiology of aortic medial disease and defective wall structure confers an ongoing risk of further dissection, aneurysmal degeneration, and rupture. Therefore, consequent control of known risk factors is crucial. In addition to age and genetic disorders such as Marfan syndrome, risk factors include lifestyle factors such as long-term arterial hypertension, smoking, dyslipidemia, and cocaine, crack cocaine or amphetamine substance use [3]. All this can be highly traumatic and stressful for affected patients, their relatives, and health care professionals.

Contemporary management of aortic dissection should be multidisciplinary and include, among other things, serial noninvasive imaging, biomarker testing, genetic risk profiling for aortopathy, blood pressure and heart rate control, lipid-lowering therapies, and repairing or replacing the damaged region of the aorta [2,3,7], with evidence that successful surgical intervention substantially improves the quality of life of aortic dissection survivors [8-10].

With regard to psychosocial factors, previous recommendations focus on patient education and the achievement of lifestyle goals. They include ensuring adherence to medical treatment, genetic counselling, smoking cessation, and other risk factor modification for atherosclerotic disease, as well as avoidance of cocaine or other stimulating drugs [2]. Furthermore, counselling aortic dissection survivors on exercise and physical activity has been described as important yet challenging, given the need to ensure avoidance of potentially damaging strenuous physical activities and contact sports, while fostering moderate intensity cardiovascular activity that may be cardioprotective in this patient cohort [11].

Initial evidence showed changes in lifestyle and emotional state following aortic dissection, with physical and sexual activity decreasing slightly, the latter mostly in relation to fear, while approximately one-third of aortic dissection survivors reported new subjective feelings of depression and anxiety [12]. However, anecdotal reports suggest that regular provision of psychosocial support to aortic dissection survivors, including psychotherapy and other interventions for psychosocial distress or mental disorders, is scarce.

Despite common clinical observations of psychosocial distress related to aortic dissection, to the best of our knowledge, there is no evidence-based tailored psychosocial (ie, psychosomatic or psychotherapeutic) intervention for aortic dissection survivors, or even systematic information regarding the need for psychosocial interventions following aortic dissection. Therefore, as a first step toward the development of a tailored psychosocial support intervention for aortic dissection survivors, we conducted a survey, addressing aortic dissection survivors, relatives of aortic dissection survivors, and professionals working with patients with aortic dissection.

The main aims of this survey were to systematically identify (1) the key topics that need psychosocial attention for aortic dissection survivors, (2) the main types of desired psychosocial interventions, (3) the expected success and both the intended and unintended effects of such interventions, and (4) the preferred timing of such interventions.

Our study is in line with recent efforts to involve patients early in the development of new interventions, which has become a key issue in biomedical research (eg, see the British Medical Journal’s Partnering with Patients initiative (“nothing about us without us”) [13]. According to this movement, partnering with patients, their families, support communities, and the public is an ethical imperative, which is essential to improving the quality, safety, value, and sustainability of health care systems.

## Methods

### Study Setting and Design

We present the results from a cross-sectional survey administered during an aortic dissection information event (Aortic Dissection Awareness Day 2017) to aortic dissection survivors and their families and relatives, as well as health professionals dealing with this condition at the University Hospital Basel.

Ethical clearance for this study was acquired from the Ethikkommission Nordwest- und Zentralschweiz (EKNZ) in Basel, Switzerland (EKNZ BASEC Req-2017-00916). Each participant provided consent that his/her responses could be used for analyses that would be reported in scientific publications.

We describe the results of our survey based on the Guidance for Reporting Involvement of Patients and the Public (GRIPP) 2, the first international guidance for reporting of patient and public involvement in health and social care research [14]. The GRIPP2 short form checklist short form accompanying this article is provided as Multimedia Appendix 1.

### Study Instrument

The questionnaire we used was a self-developed instrument compiled by an interdisciplinary team. The contents of the questionnaire, including the response categories, were compiled over the course of several meetings on the basis of pertinent publications [12,15] and information obtained during clinical encounters with patients with aortic dissection. We added open response options to ensure that respondents could provide replies beyond the preselected categories. Draft versions of the questionnaire were circulated and, based on written feedback and exchanges during another meeting, the instrument was modified until consensus was reached. This resulting questionnaire covers respondents’ sociodemographic information, topics that may require psychosocial attention for aortic dissection survivors and their relatives, types of psychosocial interventions preferred, the expected intended and unintended effects and anticipated success of such interventions, and the preferred timing of such interventions. Furthermore, we asked the respondents to indicate the percentage of aortic dissection survivors they knew that had received psychosocial support or interventions, and to specify the support or interventions. Finally, we left space for additional comments.

Answer formats were predominantly prespecified response categories that allowed for additional open responses. Other questions used a 7-point Likert-scale (“strongly disagree” to “strongly agree”), or asked for frequencies expressed as percentages.

A professional translator ensured equivalence of the French and German versions of the questionnaire. An English translation of the questionnaire is provided as Multimedia Appendix 2. The German and French versions are available from the authors on request.

### Recruitment of Respondents

During an aortic dissection information event, Aortic Dissection Awareness Day 2017, we administered a paper-pencil version of our questionnaire in German and French, enabling anonymous responses. The event was open to the public and participants included aortic dissection survivors and their families and relatives, as well as health professionals dealing with this condition. All attendees were invited to participate in the survey, without any predetermined number of participants being asked to complete the survey. We included responses from aortic dissection survivors, their families and relatives, and health professionals.

### Analyses and Statistics

We analyzed the responses by using descriptive statistics, calculating means and frequencies, and estimating 95% confidence intervals (95% CIs), based on bootstrapping procedures (1000 repetitions), to provide a measure of accuracy to our findings in terms of estimates. We did not apply the bootstrapping procedure in cases where the subgroup sample size was insufficient to calculate meaningful CIs (in these cases, no 95% CIs are provided). We provide analyses for the total sample and stratified analyses according to (1) aortic dissection survivors, (2) relatives of aortic dissection survivors, and (3) health professionals concerned with patients with aortic dissection. Open answers and comments were evaluated according to the principles of Qualitative Content Analysis [16]. Statistical analyses were performed using IBM SPSS Statistics for Macintosh (Version 21.0, IBM Corp).

## Results

### Study Sample Characteristics

A total of 41 aortic dissection survivors, their families and relatives, and health professionals participated in the study and consented to the use of their information for research purposes. In addition, 3 participants (2 partially, 1 fully) completed the questionnaire without consenting to the use of their information for research purposes. Data from these 3 subjects (7% of eligible respondents) and data from 3 noneligible respondents (who did not fall under one of the 3 subgroups surveyed in this study; 6% of all respondents) were excluded from analyses. The majority of the 41 subjects included in the study were patients with aortic dissection (n=27), followed by relatives of patients with aortic dissection (n=8), and health professionals (n=6). The characteristics of the study sample are presented in the table below (Table 1).

**Table 1:** Characteristics of the study sample.

Characteristics		Patients with aortic dissection (n=27), n (%)	Relatives of patients with aortic dissection (n=8), n (%)	Health professionals (n=6), n (%)	All subjects (n=41), n (%)
**Gender**					
	Male	21 (84)	0 (0)	4 (67)	25 (69)
	Female	4 (16)	5 (100)	2 (33)	11 (31)
	No response^a^	2	3	N/A^b^	5
**Age (years)**					
	<30	1 (4)	0 (0)	2 (33)	3 (7)
	30-39	0 (0)	1 (13)	0 (0)	1 (2)
	40-49	3 (11)	0 (0)	3 (50)	6 (15)
	50-59	7 (26)	2 (25)	1 (17)	10 (24)
	60-69	10 (37)	4 (50)	0 (0)	14 (34)
	>69	6 (22)	1 (13)	0 (0)	7 (17)
**Language**					
	German	26 (96)	8 (100)	6 (100)	40 (98)
	French	1 (4)	0 (0)	0 (0)	1 (2)
**Number of other patients with aortic dissection in contact with in a year**					
	None	17 (68)	2 (25)	1 (17)	20 (51)
	1-3 patients	8 (32)	6 (75)	2 (33)	16 (41)
	4-8 patients	0 (0)	0 (0)	0 (0)	0 (0)
	9-19 patients	0 (0)	0 (0)	0 (0)	0 (0)
	20-40 patients	0 (0)	0 (0)	1 (17)	1 (3)
	≥40 patients	0 (0)	0 (0)	2 (33)	2 (5)
	No response^a^	2	N/A	N/A	2

^a^Where participants did not respond to a question, they were not counted in the percentage calculations.

^b^N/A: not applicable.

### Key Topics

**Figure 1:**
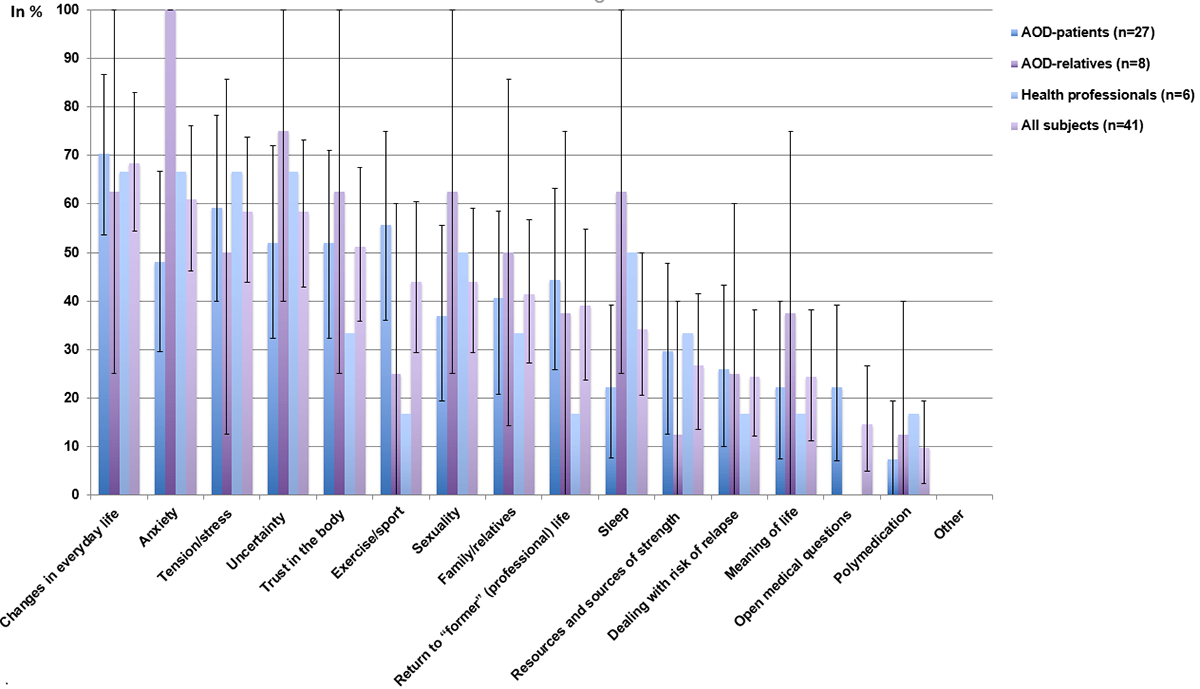
Frequency of subjects mentioning a certain concern or topic of interest to aortic dissection survivors to be addressed by psychosocial-psychotherapeutic interventions (mean and 95% CI). AOD: aortic dissection.

On average, 6 topics (95% CI 5.2-6.9; Table 2) that need psychosocial attention after aortic dissection were indicated per respondent. The most common topics (Figure 1), all chosen by more than half of the respondents, were “changes in everyday life” (28/41, 68%, 95% CI 54.5%-82.9%), “anxiety” (25/41, 61%, 95% CI 46.2%-76.2%), “uncertainty” (24/41, 59%, 95% CI 42.9%-73.2%), “tension/stress” (24/41, 59%, 95% CI 43.9%-73.8%), and “trust in the body” (21/41, 51%, 95% CI 35.9%-67.5%). The only additional topics in the free response category were “insurance issues” and “pension issues” mentioned by 1 aortic dissection survivor.

Compared to aortic dissection survivors, relatives of patients with aortic dissection indicated “anxiety,” “sexuality,” and “sleep” more often and “open medical questions” and “exercise/sport” less often (none indicated “open medical questions”). Health care professionals indicated “exercise/sport,” “return to former (professional) life,” and “open medical questions” less often than aortic dissection survivors (none indicated “open medical questions”).

### Types of Psychosocial Interventions Preferred

**Figure 2:**
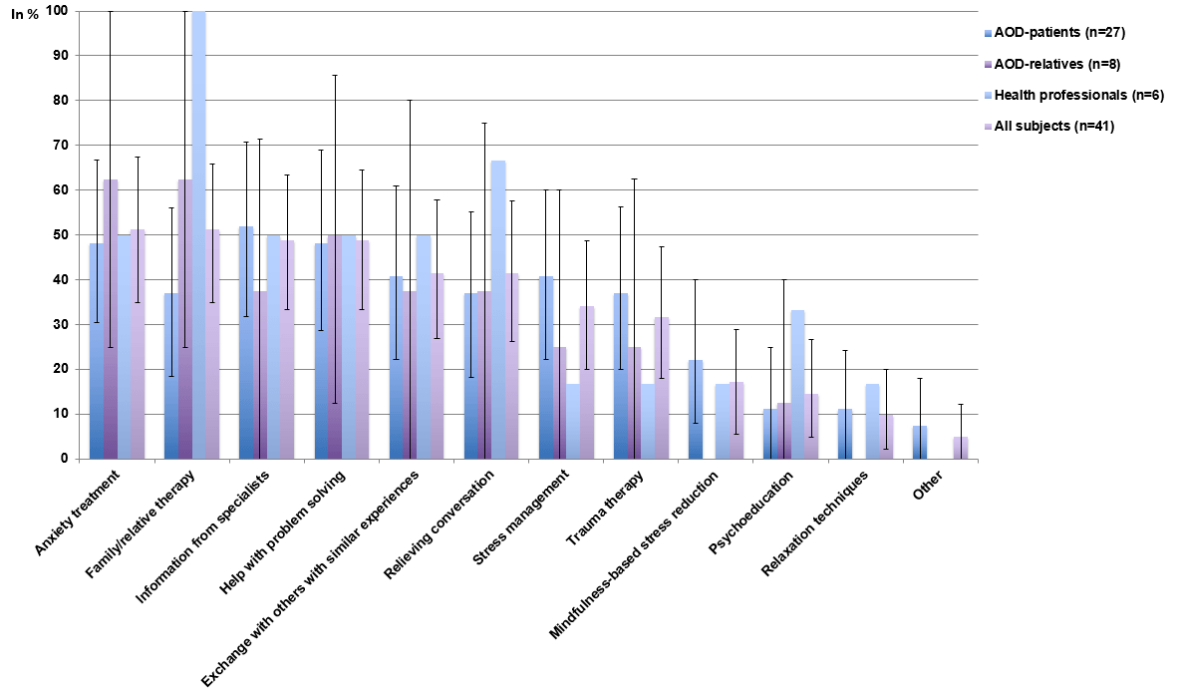
Frequency of subjects mentioning a certain type of psychosocial-psychotherapeutic intervention being of relevance to aortic dissection survivors (mean and 95% CI). AOD: aortic dissection.

On average, each respondent indicated 4 different types (95% CI 3.3-4.7) of desired psychosocial interventions for aortic dissection survivors. The most common types (Figure 2), indicated by more than half of the respondents, were “family/relative therapy” (21/41, 51%, 95% CI 35%-65.9%) and “anxiety treatment” (21/41, 51%, 95% CI 35%-67.5%). In addition, 2 aortic dissection survivors used the free response option to add “help with clarifications regarding insurance and pension issues” and “obtaining a better understanding of what residual impairment is ‘normal’ after aortic dissection.”

Compared to aortic dissection survivors, relatives indicated “family/relative therapy” more often and “mindfulness-based stress reduction” (MBSR) and “relaxation techniques” less often (not at all). Compared to aortic dissection survivors, health care professionals chose “family/relative therapy,” “relieving conversation,” and “psychoeducation” more often, and “stress-management” and “trauma therapy” less often.

### Best Timing of Intervention

**Figure 3:**
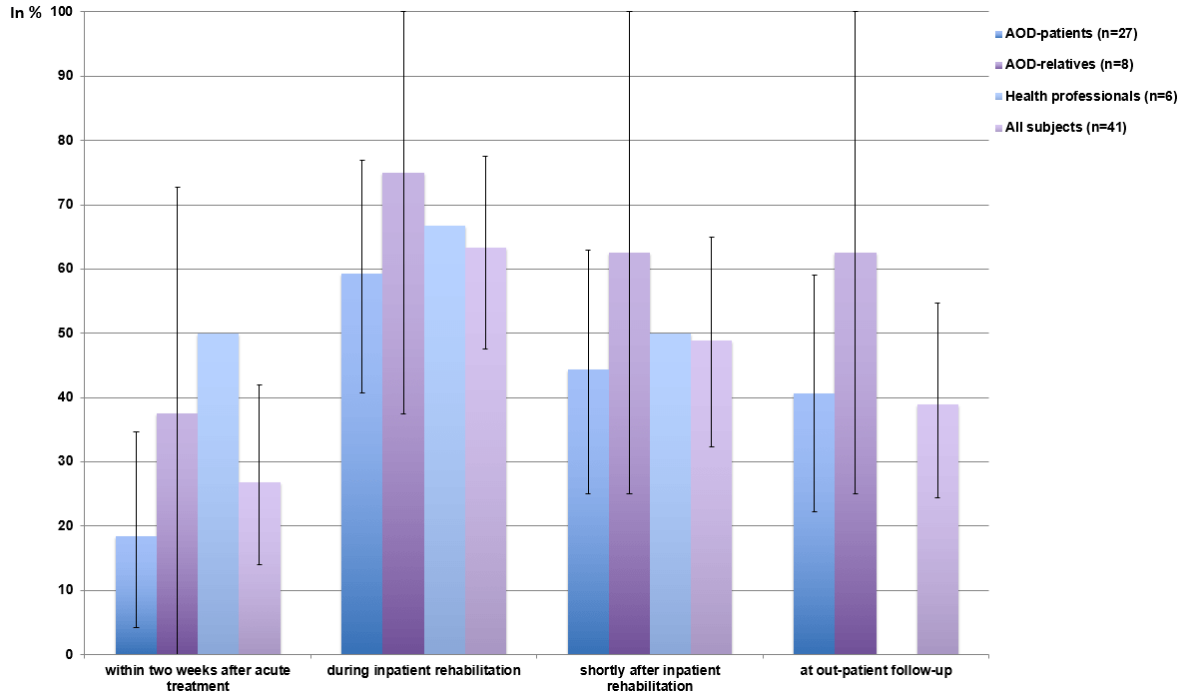
Frequency of subjects proposing a certain time as appropriate for psychosomatic or psychotherapeutic support after aortic dissection (mean and 95% CI). AOD: aortic dissection.

The most commonly chosen appropriate time to propose psychosocial support after aortic dissection was “during inpatient rehabilitation” (26/41, 63%, 95% CI 47.6%-77.5%) followed by “shortly after inpatient rehabilitation” (20/41, 49%, 95% CI 32.4%-65%), “at outpatient follow-up” (16/41, 39%, 95% CI 24.4%-54.7%), and “within two weeks after acute treatment” (11/41, 27%, 95% CI 14%-41.9%; Figure 3).

### Anticipated Success

**Figure 4:**
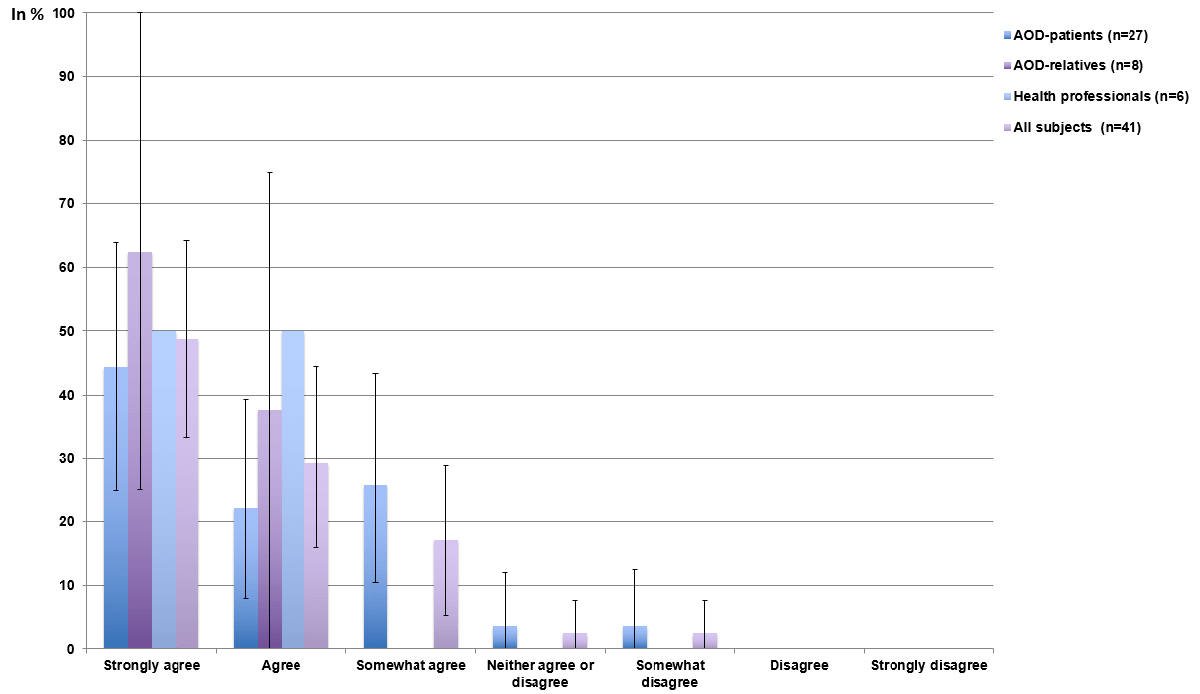
Frequency of subjects agreeing that patients would benefit from psychotherapeutic support after aortic dissection (mean and 95% CI). AOD: aortic dissection.

More than 95% (39/41) of respondents “strongly agreed” (20/41, 49%, 95% CI 33.3-64.3), “agreed” (12/41, 29%, 95% CI 15.9-44.5), or “somewhat agreed” (7/41, 17%, 95% CI 5.4-28.9) that patients would benefit from psychosocial support after aortic dissection, with relatives and health care professionals being slightly more optimistic than aortic dissection survivors (Figure 4; mean scores provided in Table 2).

### Intended Effects and Side Effects

In total, a mean of 68.6% of respondents expected that aortic dissection survivors (95% CI 61.4-76.2) would improve due to psychosocial support, while a mean of 5.2% (95% CI 2.9-7.9) expected they would be worse off. Furthermore, a mean of 5.7% of aortic dissection survivors (95% CI 1.8-10.4) expected to experience negative side effects from psychosocial support (Table 2).

Types of expected side effects were only indicated by aortic dissection survivors and included the following: “other problems predominately,” “depression,” “stress,” “the fears are greater than the support suggests,” “egocentric manifestations,” “fear of relapse,” and “to focus too much on the disease when the ability to work is restored.”

### Qualitative Findings

Answers to the open question “What else would you consider important to improve psychosomatic or post–aortic dissection psychotherapeutic support?” were sorted according to the categories identified and are provided in Table 3. The majority of responses can be subsumed under the categories “emotional support and encouragement” and “information, counsel, and assistance.”

**Table 2:** Survey results and continuous variables.

Variable	Scale	Patients with aortic dissection (n=27), mean (95% CI)	Relatives of patients with aortic dissection (n=8), mean (95% CI)^a^	Health professionals (n=6), mean (95% CI)^a^	All subjects (n=41), mean (95% CI)
Extent of agreement with “Patient would benefit from interventions”	1 (strongly disagree) to 7 (strongly agree)	6 (5.57-6.4)	6.63 (6.25-7)	6.5 (6-7)	6.2 (5.9-6.5)
Expected frequency of aortic dissection survivors that improve	Percentages	66.7 (56.9-76.4)	70 (53.8-85.5)	75	68.6 (61.4-76.2)
Expected frequency of aortic dissection survivors that decline	Percentages	5.5 (2.9-8.2)	5 (5-5)	4.2	5.2 (2.9-7.9)
Expected frequency of aortic dissection survivors with negative side effects	Percentages	9.1 (3.4-16.4)	0	0.8	5.7 (1.9-10.4)
Frequency of acquaintances receiving psychosocial treatment	Percentages	2.2 (0-6.7)	16.7 (0-50)	35	12.5 (0.8-28.9)
Number of mentioned topics of aortic dissection survivors that need psychosocial attention per respondent	Absolute frequency	5.9 (4.7-7.1)	6.8 (5.6-8)	5.5	6 (5.2-6.9)
Number of mentioned types of desired psychosocial interventions per respondent	Absolute frequency	3.9 (3.1-4.8)	3.5 (2.5-4.5)	4.7	4 (3.3-4.7)

^a^95% CI was calculated given sufficient sample size and distribution of values.

**Table 3:** Open answers to the question “What else would you consider important to improve psychosomatic or post–aortic dissection psychotherapeutic support?”

Type of respondent and response		Category
**Patients with aortic dissection**		
	Encouraging conversation	Emotional support, encouragement
	Being accompanied	Emotional support, encouragement
	That one deals with the patient intensively and really does not give up	Emotional support, encouragement
	I was supported by [*name of physician*]. That carried me through the operation and recovery	Emotional support, encouragement
	To help people who need invalidity insurance after aortic dissection, in the fight with the insurance company and the pension fund	Information, counsel, assistance
	Support with invalidity insurance	Emotional support, encouragement
	Family support	Family
	Inclusion of relatives and starting during rehabilitation	Family
	That the option is offered and that relatives can access help both now and later	Family
	With mechanical heart valve (how to deal with the sound)	Information, counsel, assistance
	That a consultation is offered as early as possible. Similar to care teams after suicide or comparable events. I had to fight to receive a psychological conversation during rehabilitation. Thereafter, psychotherapy.	Information, counsel, assistance
	Assistance in dealing with AHV/IV (pension insurance invalidity insurance)/pension fund, etc. Clarification of financial situation.	Information, counsel, assistance
	Workplace: What work can I still do?	Information, counsel, assistance
	To provide necessary information (addresses) of contacts after leaving the hospital (eg, cardiologist, psychologist, etc)	Information, counsel, assistance
	To inform patients and their relatives early regarding opportunities for support	Information, counsel, assistance
	You should go back to “everyday life” but still take care	Topics
	Not getting good sleep	Topics
	Reintegration into the work process is a challenge for indefinable reasons (difficult-to-explain symptoms)	Topics
**Relatives of patients with aortic dissection**		
	Someone who listens to you and appreciates you!	Emotional support, encouragement
	…that the relatives are involved. Too bad that this offer did not exist 5 years ago.	Family
	Patient and relatives	Family
	Show ways for the future; to reduce fear; to process the event; support with invalidity insurance	Emotional support, encouragement Information, counsel, assistance
	To reduce fear by education	Information, counsel, assistance
**Health professionals**		
	To reduce fear through better information	Information, counsel, assistance

### Already Received Psychosocial Support

The average percentage of aortic dissection survivors that have received psychosocial support or treatment known to the respondents was 12.5% (95% CI 0.8%-28.9%; Table 2).

The type of support received included “relaxation exercises,” “exercise/sport,” “nutrition,” “support from psychologist already received because of severe arthritis and claustrophobia,” “self-organized after rehabilitation,” “the family doctor provided a lot of support with conversation, etc,” and “my partner was looking for support himself.”

## Discussion

In line with current patient involvement standards and to inform the development of psychosocial interventions, this study aimed to identify the needs of aortic dissection survivors, as indicated by aortic dissection survivors, their relatives, and health care professionals.

The most common topics to be addressed with such interventions included “disturbances in everyday life,” “anxiety,” “uncertainty,” “tension/stress,” and “trust in the body.” The preferred types of interventions included “family/relative therapy” and “anxiety treatment.” The top recommended intervention timing was “during inpatient rehabilitation” followed by “shortly after inpatient rehabilitation.” Respondents anticipated that aortic dissection survivors would largely benefit from psychosocial interventions, on average expecting that approximately two-thirds would improve while only few would worsen or experience negative side effects.

Our study confirms the clinical impression that as current practice, only a minority of aortic dissection survivors receive psychosocial support, and if they do, it is limited to very few topics, such as exercise, sports, or anxiety.

Our findings are in line with and extend previous evidence on aortic dissection survivors, underlining the relevance of anxiety and uncertainty, topics related to activity, sports, and exercise, as well as sexuality after aortic dissection [12]. However, our findings also highlight hitherto unidentified topics, such as “trust in the body,” “tension or stress,” “everyday and professional life,” as well as “family and relatives.” Furthermore, the topics identified here are compatible with and expand reports on patients with unspecified life-threatening diseases in the context of intensive care units, with previous reports highlighting anxiety, uncertainty, and stress [17].

As the study comprised far fewer relatives and health professionals than aortic dissection survivors, conclusions based on comparisons between these groups’ responses need to be drawn with caution. Nevertheless, our findings suggest that while there appeared to be a large overlap of views across these groups, there were also relevant differences (eg, compared to aortic dissection survivors, relatives mentioned anxiety and uncertainty more often as topics to address, and family/relative therapy as a desired intervention).

### Strengths and Limitations

Our study has several strengths. We not only approached aortic dissection survivors and professionals, but also relatives of aortic dissection survivors, adding a perspective relevant to aortic dissection survivors’ needs and expectations regarding psychosocial interventions. Furthermore, we assessed a broad spectrum of potential intervention topics and types, complemented by open questions, thereby embracing a broad range of needs regarding psychosocial interventions after aortic dissection. Our study also has several limitations: (1) By recruiting subjects as a convenience sample on an aortic dissection information day, we cannot exclude selection bias, potentially limiting the generalizability of our findings. However, the age and gender ratio of aortic dissection survivors participating in the survey was largely comparable to previous reports [18]. (2) The sample consisted primarily of aortic dissection survivors, and included a rather small number of relatives and health care professionals. All relatives that participated in the survey and provided gender information were female, highlighting the need to approach male relatives of aortic dissection survivors in future studies. (3) Given the moderate sample size, we did not stratify analyses with regard to age group, gender, or subtypes of aortic dissection. However, there is no clear rationale as to why a certain subtype of aortic dissection should have a risk profile different than that of other subtypes.

### Implications for Research and Clinical Practice

Future studies should increase the number of respondents, including relatives of patients with aortic dissection as well as health professionals with different specializations (cardiologists, psychiatrists, etc); address additional questions, such as the preferred setting of psychosocial interventions (individual face-to-face versus group face-to-face versus online interventions); and expand the response categories to include additional topics, such as substance use.

The results of our study have important clinical implications. They guide the development of psychosocial interventions for aortic dissection survivors and their relatives in several ways: (1) the results highlighted the large number and substantial variety of issues that should be targeted, suggesting that a modular approach that allows a tailored and personalized compilation of intervention modules and techniques may be required to appropriately address diverse and complex individual needs; (2) they underscored the importance of potentially involving family and relatives and addressing related topics; (3) the results indicated potential undesired effects, such as iatrogenic fixation onto fears and the disease, hindering the focus necessary for restoration of participation in daily life and working ability; and (4) they suggested that interventions should be available along the whole disease period, from acute treatment directly after aortic dissection, to inpatient rehabilitation and the time thereafter, including outpatient follow-up.

We are currently establishing consultation-liaison psychosomatic support for patients with aortic dissection, informed by the findings reported here. To this end, we consider the individual needs of each patient and her or his family to develop interventions that fit each support-seeker best.

### Conclusions

In this study, we described the results from a survey answered by aortic dissection survivors, their relatives, and health care professionals. These results provide a basis to inform the development of tailored psychosocial interventions. Overall, patient involvement was very well perceived and feasible, suggesting that it should become common practice when developing new psychosocial interventions in cardiology and beyond. Our findings highlight a substantial need and the anticipated success of psychosocial interventions for aortic dissection survivors and their relatives, and provide the basis for the development and evaluation of therapies that could become part of state-of-the-art aortic dissection management.

## Appendix

Multimedia Appendix 1 GRIPP2 checklist.

Multimedia Appendix 2 Questionnaire.

## Abbreviations

EKNZ: Ethikkommission Nordwest- und Zentralschweiz
GRIPP: Guidance for Reporting Involvement of Patients and the Public
IPU: International Psychoanalytic University
SNSF: Swiss National Science Foundation
