# Supplementary material for: Perceived Need for Psychosocial Support After Aortic Dissection: Cross-Sectional Survey
Source: J Particip Med. 2020 Jul 6;12(3):e15447. doi: 10.2196/15447 (PMC7434062; doi:10.2196/15447)
Supplement: Multimedia Appendix 1 [file jopm_v12i3e15447_app1.docx]

**Supplementary File 1:** GRIPP2 checklist short form

| Section and topic | Item | Reported on page No |
| --- | --- | --- |
| 1: Aim | Report the aim of PPI in the study | 2 |
| 2: Methods | Provide a clear description of the methods used for PPI in the study | 2-3 |
| 3: Study results | Outcomes—Report the results of PPI in the study, including both positive and negative outcomes | 3-9 |
| 4: Discussion and conclusions | Outcomes—Comment on the extent to which PPI influenced the study overall. Describe positive and negative effects | 9-10 |
| 5: Reflections/critical perspective | Comment critically on the study, reflecting on the things that went well and those that did not, so others can learn from this experience | 10 |

*Abbreviations: GRIPP, Guidance for Reporting Involvement of Patients and the Public; PPI, patient and public involvement.*
