# Supplementary material for: Perceived Need for Psychosocial Support After Aortic Dissection: Cross-Sectional Survey
Source: J Particip Med. 2020 Jul 6;12(3):e15447. doi: 10.2196/15447 (PMC7434062; doi:10.2196/15447)
Supplement: Multimedia Appendix 2 [file jopm_v12i3e15447_app2.docx]

**Supplementary File 2**

**“Support after aortic dissection” Questionnaire**

# WHY THIS QUESTIONNAIRE?

This questionnaire was created by the Department of Psychosomatic Medicine at the University Hospital Basel to assess the need for support from psychosomatic-psychotherapeutic specialists by persons affected by aortic dissection (AOD).

We are therefore grateful if you could answer a few questions. Of course, answering is voluntary and all evaluations are anonymous.

Thank you for your help in advance!

# Personal DATA

**Your age:** ☐ under 30 years ☐ 30 – 39 ☐ 40 – 49 ☐ 50 – 59 ☐ 60 – 69 ☐ over 69 years

**Your gender:** ☐ male ☐ female

**In what capacity do you deal with aortic dissection?**

☐ I am affected by AOD myself ☐ as a relative

☐ as one of the treating physicians ☐ as a nurse

☐ as another specialist who is in professional contact with people after AOD (if the case, which specialization?): ________________________________

☐ in a different context / another capacity (if the case, which one?): ______________________________

**How many post AOD persons are you in contact with on average, per year?**

☐ none ☐ 1–3 patients ☐ 4–8 pat. ☐ 9–19 pat. ☐ 20–40 pat. ☐ over 40 pat.

# Support NEEDS

**From your point of view, what are the concerns of/topics of interest to persons after AOD with regard to support by psychosomatic-psychotherapeutic specialists?**(multiple answers are possible)

☐ changes in everyday life ☐ return to “former” (professional) life ☐ exercise/sport
☐ uncertainty ☐ trust in the body ☐ dealing with risk of relapse

☐ family/relatives ☐ tension/stress ☐ anxiety
☐ sexuality ☐ sleep ☐ purpose in life
☐ resources and sources of strength ☐ open medical questions ☐ polymedication
☐ further topics (if yes, which ones?): ________________________________

**In your view, which support interventions could be meaningful to persons post AOD?** (multiple answers are possible)

☐ relieving conversation ☐ psychoeducation ☐ information from specialists
☐ help with problem solving ☐ treatment of anxiety ☐ relaxation techniques
☐ mindfulness-based stress reduction ☐ stress management ☐ exchange with others who have had similar experiences
☐ family/relative therapy ☐ processing of traumatic experience☐ further interventions (if yes, which ones?): ________________________________

**Do you agree with the following statement? “After AOD, people would benefit from support from psychosomatic-psychotherapeutic specialists.“**

☐ ☐ ☐ ☐ ☐ ☐ ☐

strongly disagree strongly agree

**What percentage of people after AOD do you think would show improvement due to psychosomatic/psychotherapeutic support?**

____ % would probably improve.

**What percentage of people after AOD do you think would probably be worse off due to psychosomatic/psychotherapeutic support?**

____ % would probably be worse off.

**What percentage of people after AOD do you think would probably suffer from negative side-effects from psychosomatic/psychotherapeutic support?**

____ % would probably have negative side-effects.

🡺 If over 0%, which negative side-effects would you think?

__________________________________________________________________________________________

**When would be an appropriate time to propose psychosomatic or psychotherapeutic support for persons after AOD?** (multiple answers are possible)

☐ within two weeks after acute treatment ☐ during inpatient treatment

☐ shortly after inpatient rehabilitation ☐ during outpatient follow-up

**What percentage of all the post AOD persons that you know has received psychosomatic or psychotherapeutic support?**

____ % already receives psychosomatic or psychotherapeutic support.

🡺 if more than 0%, what type of support?

__________________________________________________________________________________________

# WHAT ELSE would you consider important to improve psychosomatic or post-aod psychotherapeutic Support?

I think it’s important that: ... __________________________________________________________________________________________

__________________________________________________________________________________________

__________________________________________________________________________________________

# Agreement to use of answers for Research purposes

☐ **I agree that my data may be used anonymously for research purposes.**

# Thank You!

**Thank you for your time and for answering these questions, in the name of the Department of Psychosomatic Medicine**

____________________________ _________________________________

Prof. Dr. med. R. Schäfert Prof. Dr. med. S. Hunziker Schütz

Chief Physician, Dept. of Psychosomatics Deputy Chief Physician, Dept. of Psychosomatics,

USB Head Medical Communication, USB
